# Automatic Segmentation of Abdominal Aortic Aneurysm From Computed Tomography Angiography Using a Patch-Based Dilated UNet Model

**DOI:** 10.1109/access.2025.3533417

**Published:** 2025-01-23

**Authors:** MERJULAH ROBY, JUAN C. RESTREPO, HAEHWAN PARK, SATISH C. MULUK, MARK K. ESKANDARI, SEUNGIK BAEK, ENDER A. FINOL

**Affiliations:** 1 Department of Mechanical Engineering, The University of Texas at San Antonio, San Antonio, TX 78249, USA; 2 LG Electronics, Gasan Research and Development Campus, Seoul 08592, South Korea; 3 Department of Thoracic and Cardiovascular Surgery, Allegheny Health Network, Allegheny General Hospital, Pittsburgh, PA 15212, USA; 4 Feinberg School of Medicine, Northwestern University, Chicago, IL 60611, USA; 5 Department of Mechanical Engineering, Michigan State University, East Lansing, MI 48824, USA

**Keywords:** Abdominal aortic aneurysm, deep learning, image segmentation, computed tomography imaging, NURBS

## Abstract

Abdominal Aortic Aneurysm (AAA) is still a socially relevant public health challenge, evidenced by an 82.1% increase in associated fatalities from 1990 to 2019 (with 172,427 deaths in 2019 alone). In a clinical setting, computed tomography angiography (CTA) is the imaging modality of choice for monitoring and/or presurgical planning of AAA patients. However, manual segmentation of CTA images is labor intensive and time consuming. Hence, there is a growing need for automated segmentation algorithms, particularly when these influence treatment planning. The deep-learning pipeline proposed in this work is designed to automatically segment AAA CTA images. The framework adapted a fully developed patch-based dilated modified U-Net model, which shows remarkable efficiency in accurately delineating AAA regions within the CTA scans. During the prediction phase, the deep learning architecture demonstrates exceptional speed, requiring 17 ± 0.02 milliseconds per frame to generate the final segmented output. Building upon this work, we included the application of Non-Uniform Rational B-Splines (NURBS) to enhance the segmentation process. This advancement is essential in addressing the critical need for clinical accuracy in medical image segmentation. NURBS enables the creation of continuous curves that seamlessly conform to the intricate contours of anatomical structures, offering a significant improvement in segmentation accuracy. Through the integration of advanced deep learning architectures and the precision of NURBS for segmentation refinement, coupled with the fast processing time and accurate segmentation, the the proposed model represents a promising clinical tool that can be used in the clinical management of AAAs.

## 1. INTRODUCTION

An Abdominal Aortic Aneurysm (AAA) is a serious condition in which the distal end of the main artery of the body, the aorta, dilates abnormally. This dilation can be life-threatening if the abdominal aorta ruptures. Various factors contribute to its development, including aging, smoking, high blood pressure, plaque buildup (atherosclerosis) and male gender [1]. In its early stages, an AAA often does not show symptoms, making it challenging to detect without proper screening. However, as the aneurysm grows, it can cause severe abdominal or back pain and eventually rupture if left untreated. A ruptured AAA is a medical emergency and is a nearly fatal event. In clinical practice, the maximum AAA diameter is the sole predictor of AAA growth and is used to assess rupture risk [2]. The current guidelines for AAA repair for men are a maximum diameter ≥ 55 mm and ≥ 50 mm for women [3]. When choosing the appropriate size of the endovascular graft for AAA repair, the interventionist estimates the neck diameter and common iliac artery diameters, among other measures [4].

AAAs are typically diagnosed using imaging methods such as ultrasound, CTA exams, or magnetic resonance imaging (MRI) exams. CTA plays an important role in the diagnosis and planning of AAA treatment, as it provides highly detailed, three-dimensional images of the aorta and nearby soft tissues. CTA images are used to accurately measure the size and shape of an aneurysm, and detailed dimensions needed to make decisions about interventional treatment. These images can be segmented semiautomatically or automatically to identify specific regions of interest (ROI). Semi-automatic methods often require initial guidance through seed points and commonly use deformable models or active shape models. Efforts have been made towards achieving fully automated AAA segmentation. In particular, Pham and Golledge [5] introduced a novel method called Geostatistically Constrained Fuzzy CMeans (FCM), which integrates spatial covariance from geostatistics theory into fuzzy c-means. This integration allows for the automatic segmentation of AAAs. Similarly, Dehmeshki et al. [6] proposed a computer-aided detection system that automates AAA segmentation. The system initially identifies the aortic lumen and subsequently uses geometric and morphological characteristics to assess the presence of an aneurysm.

With the goal of identifying the extent of the AAA, Kurugol et al. [7] predicted the outer wall contour in non-contrastenhanced CTA images using anatomical localization, circular Hough transform, and 3D level set segmentation. Shang et al. [8] employed a semiautomated approach to track the AAA outer wall through isointensity contours in CTA images. De Bruijne et al. [9] proposed manual delineation followed by automatic segmentation using a statistical shape model in CTA images. Wang et al. [10] extended a geodesic active contour method for AAAs in MRI. Herment et al. [11] proposed a 2D deformable surface model to segment the thoracic aorta in MR images. Xie et al. [12] introduced an algorithm employing anatomical localization and cylindertracking in non-contrast-enhanced CTA images of the thoracic aorta. Olabarriaga et al. [13] utilized a discrete deformable model based on grayscale level information for outer wall segmentation in CTA. Adame et al. [14] developed an algorithm to detect the inner and outer wall contours of the non-aneurysmal thoracic aorta and carotid arteries in high-resolution MR images using geographic model matching. Similarly, Isgam et al. [15] proposed a multi-atlas-based segmentation method for non-contrast CTA images of the thoracic aorta. Lee et al. [16] introduced a graph search-based approach with manual control points for the identification of the AAA outer wall in CTA. Raman et al. [17] developed an algorithm defining the outer wall contour for normal thoracic and abdominal aortas in contrast-enhanced CTA images. Bustamante et al. [18] introduced an atlas-based segmentation method using MR images of the thoracic aorta across cardiac cycle phases. Zhuge et al. [19] proposed an automated level-set segmentation technique requiring an appropriate initial contour derived from grayscale levels and the lumen region.

Before the integration of deep learning techniques, the aforementioned methodologies effectively represented the basis of all segmentation methods. Although they show promising performance, their optimization for improved accuracy requires additional adjustment of the parameters. Enhancing these methodologies, particularly through the integration of deep learning methods, could yield substantial improvements in accuracy and efficiency in aortic segmentation tasks. The present work introduces an innovative auto-mated segmentation technique to identify the AAA lumen and outer wall boundaries. This approach ensures exceptional reliability and effectiveness, distinctly eliminating the need for user intervention without compromising precision. The method does not require specialized AAA knowledge or specific segmentation software. It yields high accuracy and reliability due to a carefully trained dataset, ensuring precise results. The segmentation methodology establishes a solid foundation for comprehensive biomechanical assessments of AAAs and exceeds the speed of conventional segmentation methods. By enabling prompt and exhaustive evaluations, the proposed approach significantly enhances the precision and effectiveness of AAA ROI identification. Notably, the integration of dilation convolutional models and convolutional neural network (CNN) patch-based segmentation outper-forms preexisting solutions, affirming the advancement in state-of-the-art AAA segmentation tasks.

In conjunction with the CNN patch-based segmentation model, we developed a user-interactive tool for manual refinement of medical image segmentation. The segmentation results are obtained from the automatic segmentation model, which is essential for accurate and reliable diagnostic and therapeutic procedures. To further enhance its utility in clinical settings, we incorporated a NURBS-based user interactive tool, which allows for segmentation refinement to meet specific clinical accuracy requirements. The use of NURBS adjustments in the tool represents a sophisticated approach to improving medical image segmentation with the intuitiveness of manual adjustments and the mathematical accuracy needed for clinical precision. If required, this user-interactive tool can be employed for complex anatomical cases, ensuring adaptability and precision in diverse clinical scenarios.

## II. RELATED WORK

Within the domain of medical image segmentation, the patch-based dilated U-Net architecture represents an important advancement. However, its significance requires contextualizing it against the preceding methodologies employed in similar tasks. Freiman et al. [20] presented an iterative graph-cut algorithm focusing on AAA thrombus segmentation, while Lee et al. [16] relied on a 3-D graph search technique based on a triangular mesh for thrombus segmentation. The major limitation of these methods is the inconsistency in achieving high segmentation accuracy, coupled with their reliance on specific datasets, thereby limiting their robustness for clinical deployment.

Despite the limitations of prior approaches, there is a specific need for automated segmentation tools that can revolutionize medical image analysis, particularly to address the challenges associated with AAA segmentation. The Dice Similarity Coefficient (DSC) is typically used as the benchmark metric to compare these tools. Chandrashekar et al. [21] employed deep learning segmentation techniques to automate the segmentation of pathological blood vessels, specifically aortic aneurysms in CTA images. Their approach achieved a DSC score of 0.887 ± 0.005 at low resolution and 0.932 ± 0.007 at high resolution, demonstrating remarkable accuracy. Similarly, Lyu et al. [22] conducted a comparative study between attention-based Residual U-Net (ARU-Net) [23] and Context Aware Cascaded U-Net (CACU-Net) [24]. Both models yielded a DSC score of 0.916 ± 0.028 and 0.916 ± 0.029, respectively, with CACU-Net exhibiting slightly superior performance. Kim et al. [25] utilized a CNN-based segmentation developed by Zhang et al. [26] to extract lumen and outer wall geometry from 3D CTA scans, achieving a DSC score of 0.866 ± 0.044. Furthermore, Fantazzini et al. [27] developed a deep learning pipeline to automatically segment the aortic lumen in CTA scans from the ascending aorta to the iliac arteries, yielding a DSC score of 0.92 ± 0.01 and a Jaccard coefficient of 0.869 ± 0.037, underscoring both accuracy and efficiency. Subsequently, Brutti et al. [28] employed a similar methodology, achieving a mean DSC score of 0.89 in segmenting CTA images with a focus on the lumen and thrombus.

While these deep learning-based approaches represent substantial progress in AAA segmentation, they have not been applied to large and complex AAA cases, indicating the need for an improved method to address such clinically relevant aneurysms.

## III. METHODOLOGY

The segmentation of AAA images sized 256 × 256 pixels has been accomplished using the U-Net with a patch-based dilated model architecture on a GPU computing platform. While preserving the fundamental parameters and architectural components of the original implementation, the proposed approach integrates patches and dilated convolutions within the U-Net model. The utilization of dilation rates effectively augments the receptive field of the convolutional filters without an equal increase in parameters. This enhancement is achieved by introducing gaps between values within the filters, as elucidated by Yu and Koltun [29] By leveraging this technique, the proposed model acquires a more expansive contextual understanding, which is imperative for precise segmentation tasks, particularly in scenarios that require the analysis of extensive image regions.

### A. PATCH-BASED DILATED U-NET MODEL DESIGN

The 2D U-Net architecture, evolving from Ronneberger et al.’s U-Net model [30], specializes in image segmentation, particularly in medical images. It showcases an encoder-decoder structure and, more importantly, a ‘U’, enabling detailed feature extraction and high-fidelity output generation. In the U-Net architecture, dilation rates play an important role in how the network processes the image through its convolutional layers. These rates determine the gaps between the elements (or pixels) in the convolutional filters. The higher the dilation rate, the farther apart the elements are, which significantly influences how much of the image each filter considers.

**FIGURE 1.**
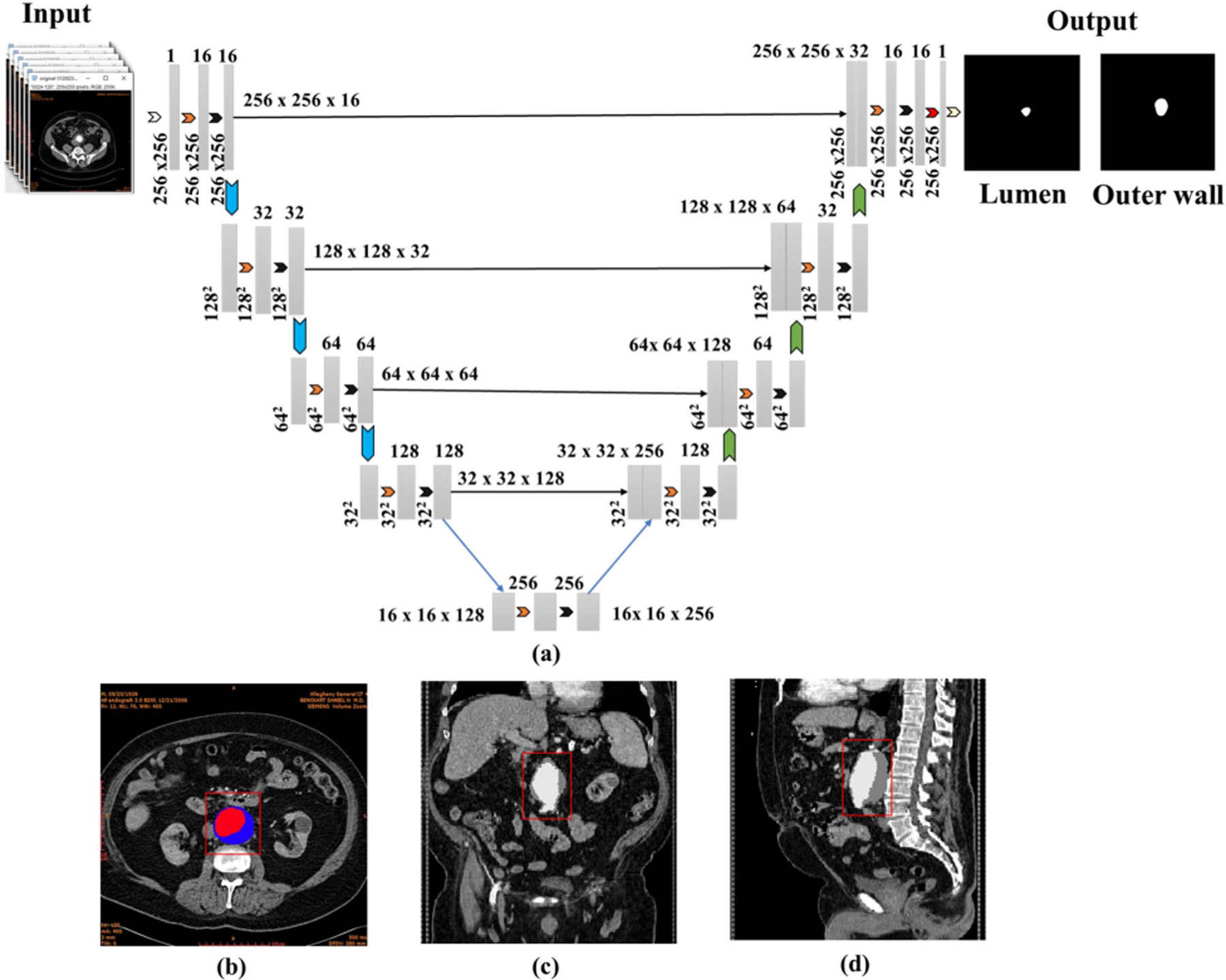
(a) Overall architecture of the patch-based dilated U-Net model; (b) Exemplary binary masks overlaid on an AAA image; (c) and (d) Reconstruction and visualization of the AAA in the coronal and sagittal planes.

The dilation rates vary strategically across layers in the proposed U-Net’s design. Initially, in the early layers of the network, a dilation rate of 1 is used. This translates into the filters closely inspecting each pixel without any gaps. However, as the network progresses deeper into subsequent layers, it strategically employs dilation rates of 2, 3, and reverts to 1, sequentially. Using these varying dilation rates, the network expands its field of view over the image. In essence, it captures information from broader areas in later layers, allowing it to gather details from larger regions of the input data. The approach is particularly beneficial for precise feature extraction, as it enables the network to perceive and comprehend intricate patterns and structures within the image. In addition to its architectural components, the methodology integrates patch-based processing, which is a technique that subdivides large image stacks into smaller, manageable patches. These patches undergo individual processing, enabling efficient analysis and segmentation of large datasets. This approach facilitates comprehensive analysis while managing computational complexity, which is beneficial in medical imaging applications with large image data sets. Furthermore, data augmentation strategies are employed to expand the training dataset and enhance model generalization. These augmentation techniques encompass various transformations, such as rotations, shifts, and zooms, implemented through the ImageDataGenerator from TensorFlow’s Keras library. Augmentation aids in reducing overfitting by exposing the model to varied representations of the training data while improving its robustness in handling unseen data during inference. The methodological aspects of the 2D U-Net’s patch-based dilated model collectively contribute to the model’s precision and scalability in medical imaging tasks. Figure 1(a) of the AAA segmentation pipeline shows the patch-based dilated UNet model, the resulting output being the lumen and outer wall boundaries of the AAA. Figure 1(b) illustrates the overlaid binary mask on the initial CTA images, while Figures. 1(c) and 1(d) provide a visual representation of the coronal and sagittal planes, respectively. The segmented images were also used to create a volume mesh using the in-house script AAAMesh [31] for visualization purposes.

### B. THE USER-INTERACTIVE NURBS-BASED TOOL

We developed a user-interactive tool based on NURBS [32], as accurate segmentation of medical images is a critical step in numerous diagnostic and therapeutic procedures. The method was implemented with an interactive Python application developed using Tkinter for the graphical user interface (GUI). This tool allows users to load CTA scan images along with their corresponding segmentation masks, providing functionalities to navigate through the image stack, and manually adjust segmentation boundaries using interactive points to the contours for refined segmentation. This immediate interaction is critical for medical professionals seeking to adjust segmentation in real time, ensuring that the refined masks accurately represent the anatomical structures of interest. The core mathematical concepts are image normalization and coloring, with a range of −400 to 1000, which can be adjusted based on the imaging modality and the regions of interest. The creation of a NURBS curve from control points derived from the sampled contour points or modified by the user involves several mathematical processes, including calculating the knot vector and evaluating the curve at a set of parameter values (delta). Equation (1) was used to calculate the NURBS curves,

$$c\left(t\right)=\frac{\sum_{i=0}^{n}N_{i,p}\left(t\right)w_{i}P_{i}}{\sum_{i=0}^{n}N_{i,p}\left(t\right)w_{i}}$$

where:

- $c\left(u\right)$ is the point on the NURBS curve at the parameter u.
- $N_{i,p}\left(u\right)$ is the i-th B-spline basis function of degree p.
- $w_{i}$ is the weight associated with the i-th control point.
- $P_{i}$ is the i-th control point.
- u is the parameter that varies from 0 to 1 along the length of the curve.

The NURBS tool allows users to manually refine segmentation by manipulating points in the contours and augmenting them with new points. Moreover, it integrates an advanced technique for enhancing existing segmentation masks with spline interpolation, yielding anatomically precise executions. If necessary, these refined capabilities can be used to achieve greater accuracy; otherwise, the predicted results can be utilized. This dual functionality ensures that the tool adapts to the specific needs of each case, whether requiring careful manual adjustment or relying on the initial automated predictions. The approach could help a user make informed decisions regarding the effect of automated predictions or the use of NURBS refinement for segments that require revision of the subsequent treatment recommendations. Compared to the previously developed in-house algorithm, AAAVasc [4], the NURBS-enhanced model achieved a 90% reduction in processing time.

### C. DATASET DESCRIPTION

Following approval of a human subjects research protocol by the Institutional Review Boards of Allegheny Health Network and Northwestern Memorial Hospital, we retrospectively collected pre-operative CTA exams for 22 AAA patients (18 with non-complex AAAs and 4 with complex AAAs). The images of these exams (1385 in total) were used for the evaluation performance of the proposed segmentation model. The ground truth used for this evaluation was the manual segmentation performed by medical experts. The differences in the segmentation results obtained by two different trained users are referred to as interobserver segmentation. Training and testing of the model were performed using CTA images from 68 alternate AAA patients (4268 in total), which were collected previously as part of a different human subjects research study.

Before a deep learning model can learn, the data should be prepared properly. This step helps the model to understand the information and make the correct predictions. Organizing and refining the data is crucial for the model to work with reasonable accuracy. Numerous techniques are employed to refine raw data and facilitate optimal model performance. One such fundamental technique is normalization, which matches input data to a uniform scale, helping ease model training and bolstering accuracy by aligning pixel values within a standardized range. We used normalization to help increase the model’s accuracy and decrease the computational processing time. Contrasting methodologies have been explored in previous works, such as the use of Histogram Equalization (HE), as demonstrated in the work of Atefeh et al. [33]. Although HE has shown promise in image enhancement, it is not devoid of limitations. Its application often introduces visual artifacts, including noise intensification, a washed-out effect, and issues with brightness shift, which impact image quality. The inherent trade-offs associated with HE, such as level saturation and over-/under-improvement, can lead to visual degradation [35, 36]. Additionally, in the pre-processing pipeline, the images were resized from their original 512 × 512 resolution to a reduced 256 × 256 resolution, to expedite the deep learning model training process. Larger images require neural networks to process significantly more pixels, subsequently extending the training time required by the architecture. The downsizing of the images not only accelerates the learning process, but also assists in managing computational resources without compromising the learning capacity of the model. The model was trained using the University of Texas at San Antonio’s ARC high-performance computing cluster, which includes nodes with two CPUs (each with 20 cores, totaling 40 cores per node), 384 GB of RAM, and two Nvidia V100 GPU accelerators per node.

## IV. RESULTS

### A. SEGMENTATION OF THE LUMEN AND OUTER WALL BOUNDARIES

**FIGURE 2.**
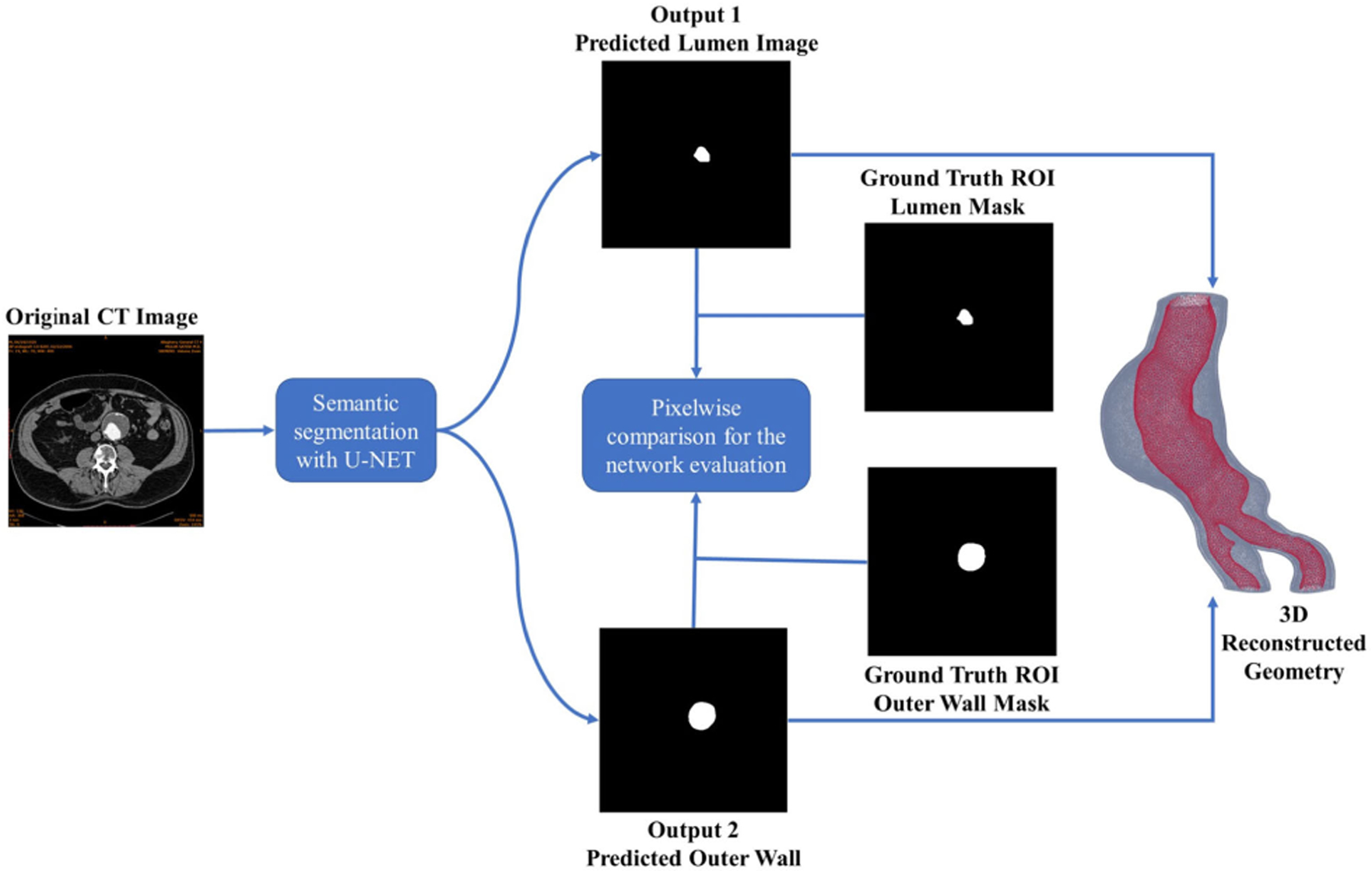
Image segmentation pipeline, which includes identification of the lumen and outer wall ROIs, followed by volume mesh generation.

Accurate boundaries for the lumen and outer wall were established using the patch-based dilated U-Net model trained with 4268 contrast-enhanced CTA images and validated with an additional 1385 images. Figure 2 illustrates the segmentation pipeline outlining the anatomical components of interest. The segmented images were used to create a volume mesh. This process facilitated the conversion of segmented boundaries into a comprehensive 3D representation, essential for any desirable biomechanical assessment via finite element analysis. The resulting 3D mesh encapsulates the intricate features of the lumen and outer wall ROIs, providing a foundational structure for advanced biomechanical analysis.

**FIGURE 3.**
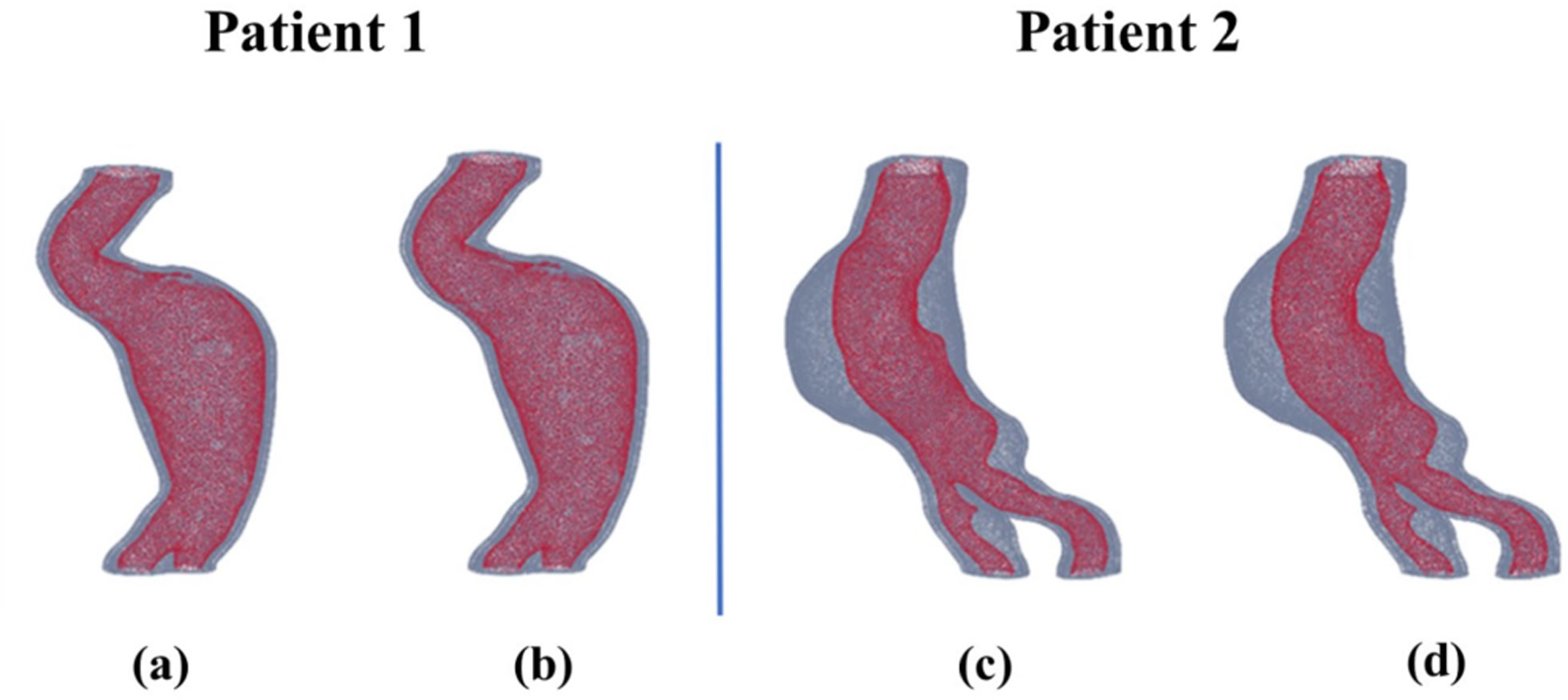
Volume meshes generated from the ground truth [(a) and (c)] and the predicted [(b) and (d)] image segmentations.

A comparison of visual representations was made, specifically analyzing the geometries generated from the ground truth (manual segmentation performed by medical experts) and the predicted segmentations. To evaluate the segmentation performance, the predicted segmentations were compared with the ground truth obtained from images previously segmented using AAAVasc. Figure 3 illustrates representations of the volume meshes generated from the original and predicted segmentations. Figures 3(a) and 3(c) are the ground truth volume meshes of patients 1 and 2, respectively, while Figures. 3(b) and 3(d) are the predicted volume meshes of patients 1 and 2.

During segmentation, the model operates using the binary cross-entropy (BCE) and the dice coefficient (DC) loss function to delineate the lumen and outer wall of the AAA. Unlike categorical cross-entropy (CCE) in multiclass segmentation tasks, BCE focuses on pairwise classifications within the broader set of classes. This tailored approach allows the model to discriminate specifically between the lumen and outer wall boundaries. By emphasizing crucial distinctions without the complexity of simultaneous consideration of all classes, BCE simplifies the learning process. The application of BCE in multiclass segmentation tasks enables the model to make finer distinctions between classes, significantly enhancing the precision and accuracy in identifying segments. Specific pairwise comparisons facilitate focused learning, contributing to an improved segmentation performance. In multiclass segmentation tasks, the use of BCE for pairwise classifications within the broader set of classes offers distinct advantages over CCE. BCE enables focused learning by delineating specific binary comparisons, such as discerning the lumen and outer wall. This approach simplifies the learning process and emphasizes critical distinctions without the complexity of simultaneous consideration of all classes. Using BCE helps to make finer distinctions between classes, improving the accuracy with which the segments are identified. This strategy shows the efficiency of employing BCE for specific pairwise comparisons within multiclass segmentation tasks, demonstrating its practicality and potential to enhance segmentation performance.

### B. PERFORMANCE METRICS FOR SEGMENTATION ASSESSMENT

**FIGURE 4.**
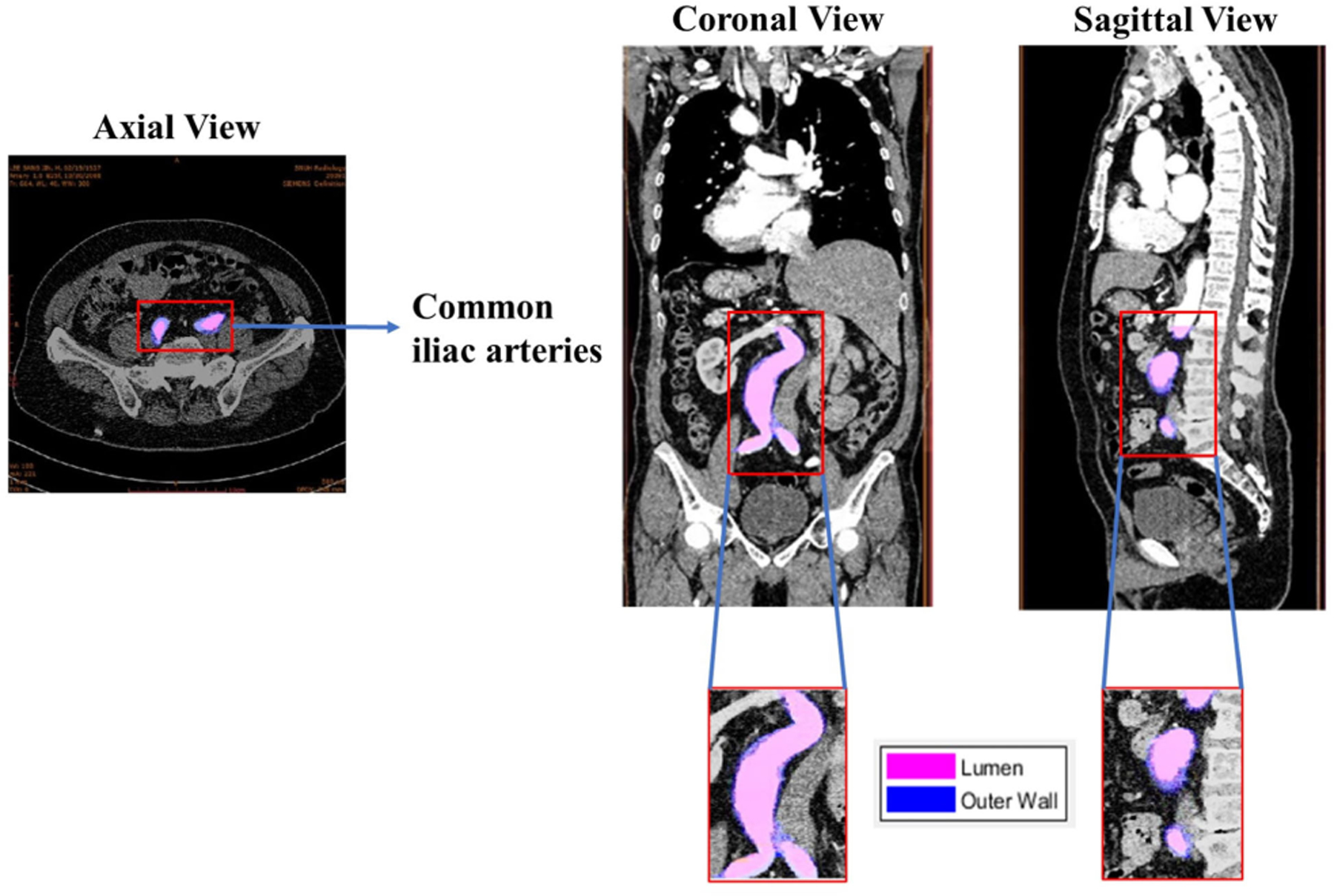
Visualization of the common iliac arteries, lumen, and wall regions of interest in the axial, coronal, and sagittal planes of an exemplary AAA.

Visualization plays an important role in understanding anatomical structures. The algorithm presented in this work is specifically developed for the comprehensive processing and visualization of CTA image datasets. The pipeline entails initial image reading and aggregation into a coherent 3D volume, serving as a representation of the entire image collection. The core functionality involves the extraction of three fundamental anatomical planes —axial, coronal, and sagittal— each important for achieving distinct visualization and analysis in medical imaging. In Figure 4, the axial view of an exemplary AAA shows the segmented common iliac arteries, while the coronal reconstruction illustrates the overlapped lumen and wall regions. Furthermore, the sagittal view shows the overlapping lumen and wall regions, while providing information on other anatomical features.

The metrics shown in Table 1 indicate a substantial overlap between the predicted results segmented by the model and the ground truth. For the 18 non-complex AAAs, the model yielded accuracies of 99.95% and 99.92% for the lumen and outer wall segmentation, respectively. This outcome exemplifies the model’s precision in correctly identifying and delineating the AAA boundaries of interest. The model’s performance was also evaluated with the 4 complex AAAs with high tortuosity (the metrics for these complex cases are also included in Table 1). It maintained its high accuracy levels, achieving 99.95% for the lumen segmentation and 99.94% for the outer wall segmentation. With sensitivity scores greater than 94% in all cases, the model demonstrates its ability to detect true positives accurately. Precision scores, which are approximately 96% for the non-complex cases and nearly 98% for the high tortuosity cases, emphasize the model’s precision in correctly identifying the targeted structures without false positives, showcasing its strong performance with challenging anatomy. The values of Matthew’s Correlation Coefficient (MCC), ranging from 0.9566 to 0.9736, indicate a strong correlation between the predicted and original classification. DSC values, which are in the range of 0.9562 to 0.9737, further reinforce the model’s robustness in overlapping segmentations. In addition, the Intersection over Union (IOU) scores, varying from 0.9175 to 0.9492, are indicative of the accuracy and reliability of the model, corroborating its effectiveness in precise segmentation.

### C. QUANTITATIVE ANALYSIS OF AAA SEGMENTATION ACROSS MULTIPLE CROSS-SECTIONS

**FIGURE 5.**
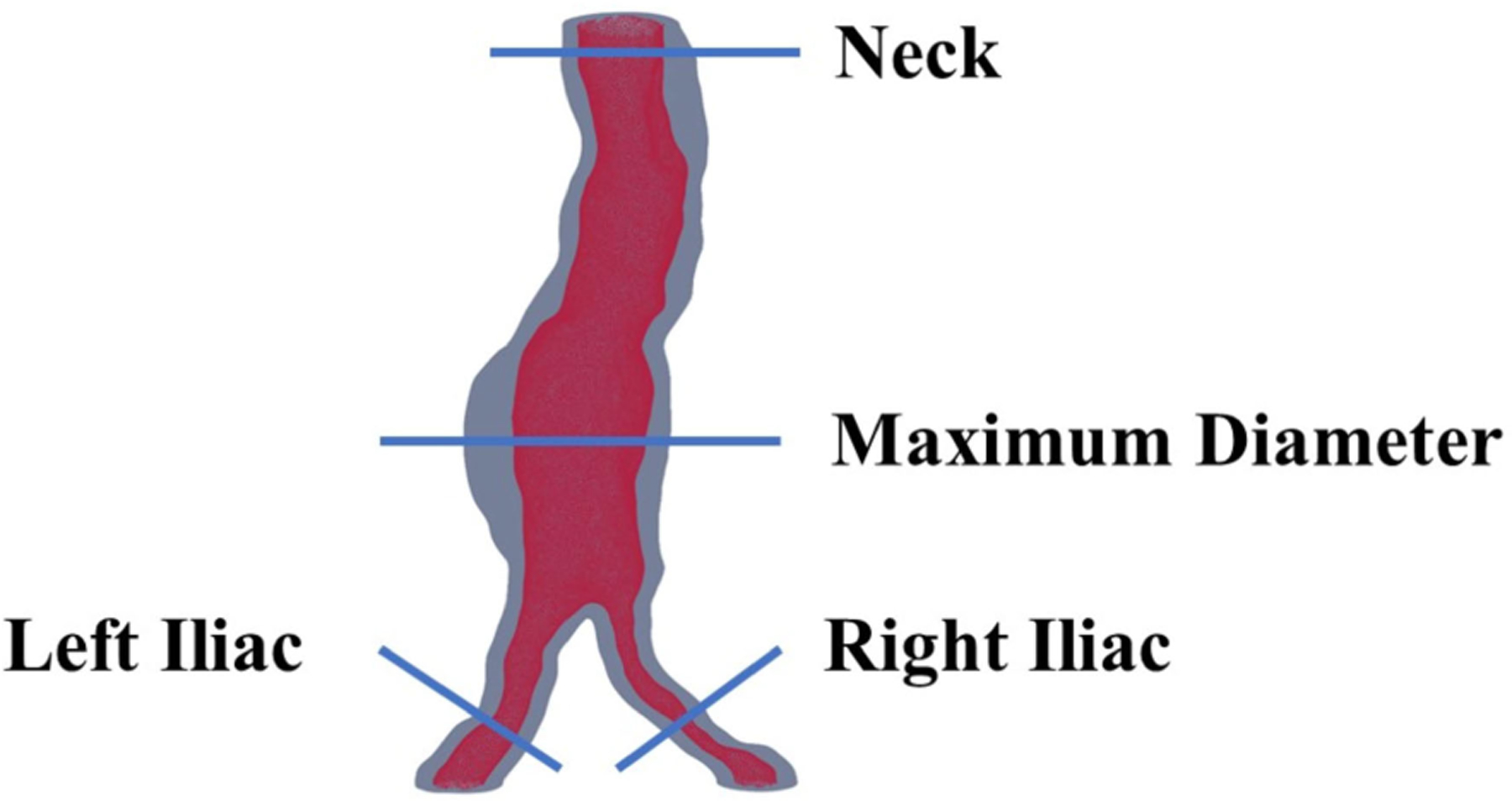
Four cross sections of interest to evaluate model performance.

Figure 5 illustrates four distinct cross sections essential for clinicians to assess AAA growth and the sizing of the endovascular graft for AAA repair. Assessment of segmentation at various cross sections is critical for precise measurement and subsequent clinical decision-making. Therefore, the evaluation of the model’s accuracy and reliability was conducted through quantitative assessments of the lumen area and hydraulic diameter at three cross sections: the neck, and the left and right common iliac arteries. This evaluation used three different segmentation techniques: expert medical segmentation, interobserver segmentation, and predictive algorithmic segmentation, which ensured a thorough validation of the model’s capabilities.

**FIGURE 6.**
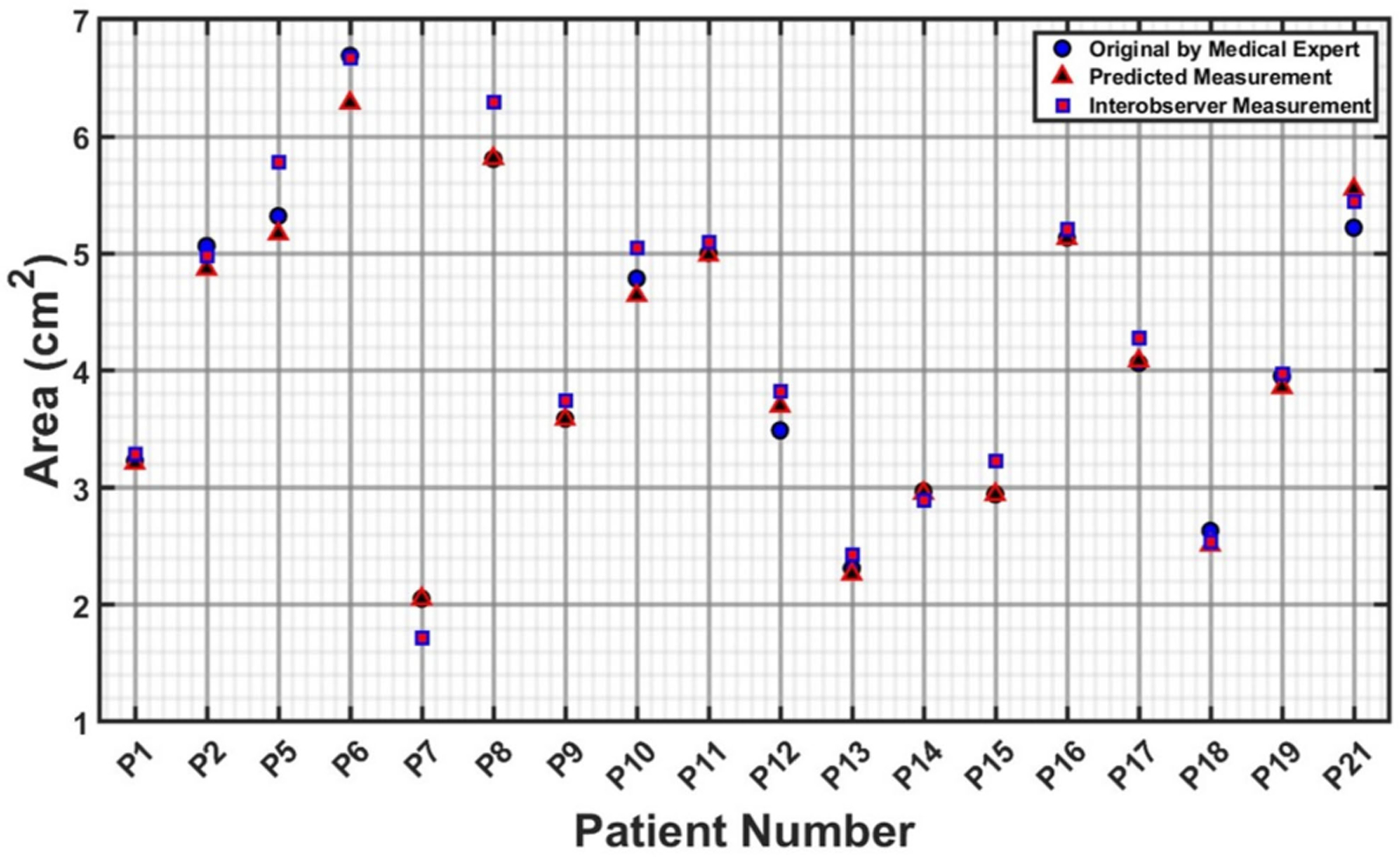
Comparative analysis of neck lumen area (in cm^2^).

**FIGURE 7.**
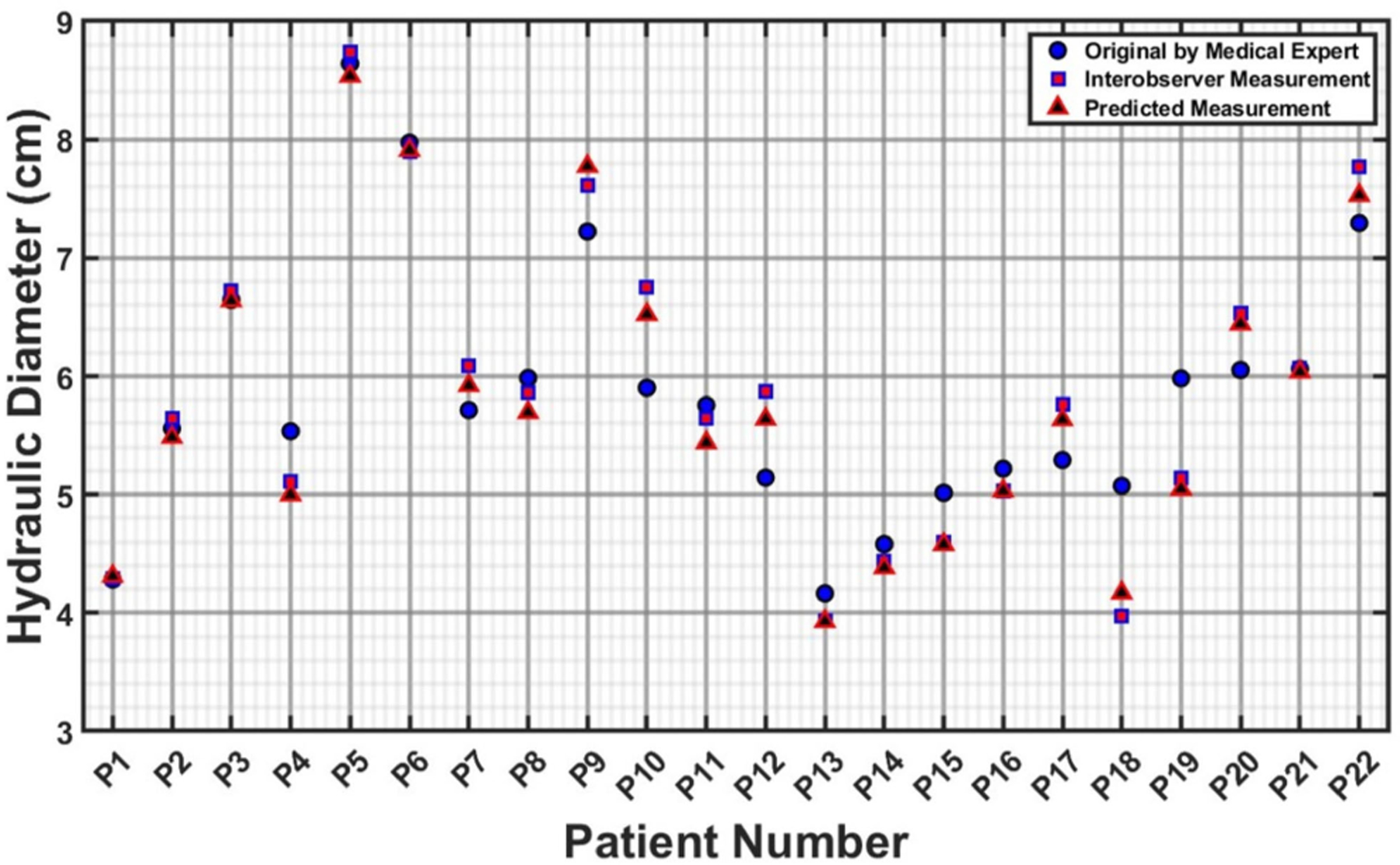
Comparative analysis of maximum hydraulic diameter (in cm) measured using the outer wall boundary.

**FIGURE 8.**
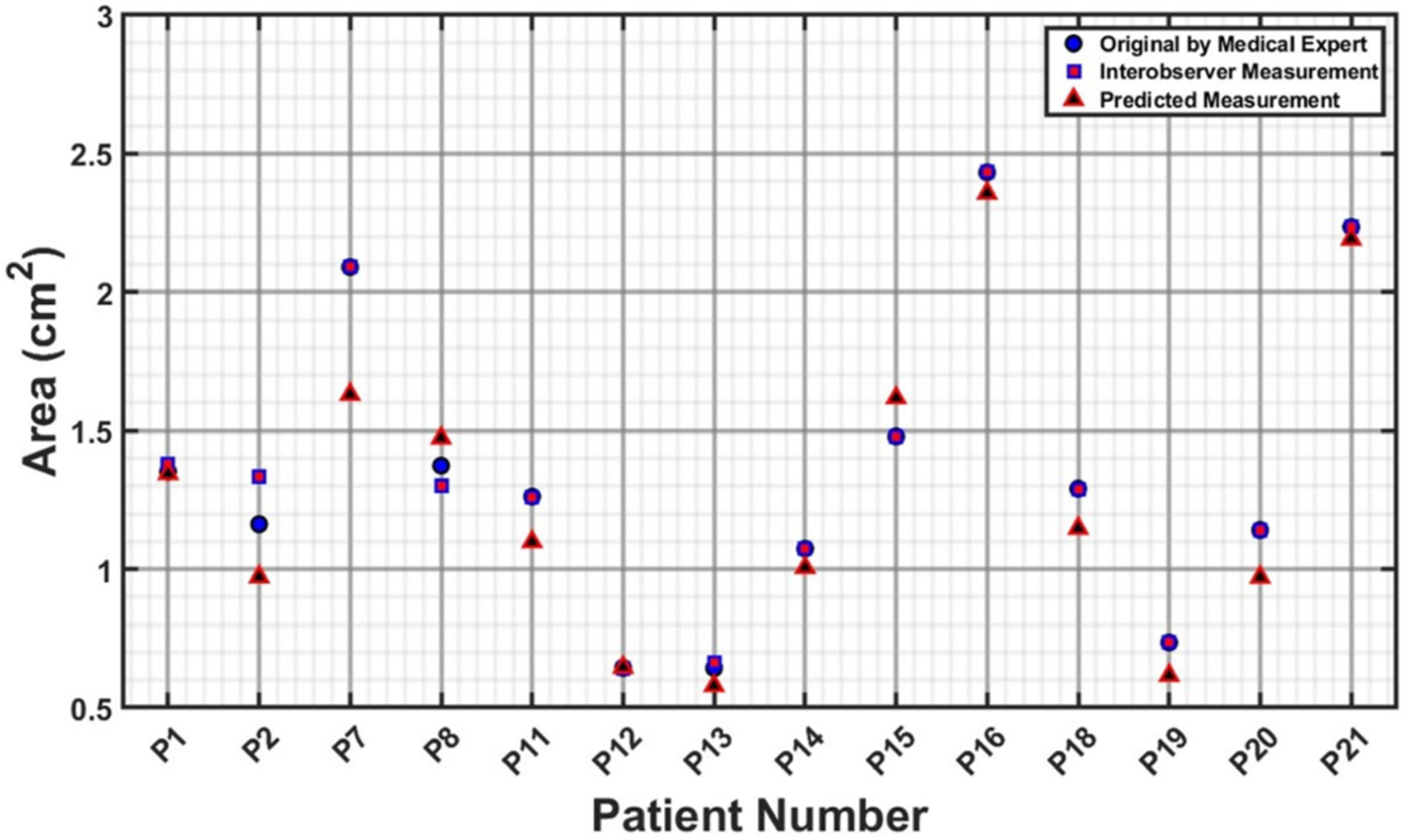
Comparative analysis of lumen area of the left common iliac artery (in cm^2^).

**FIGURE 9.**
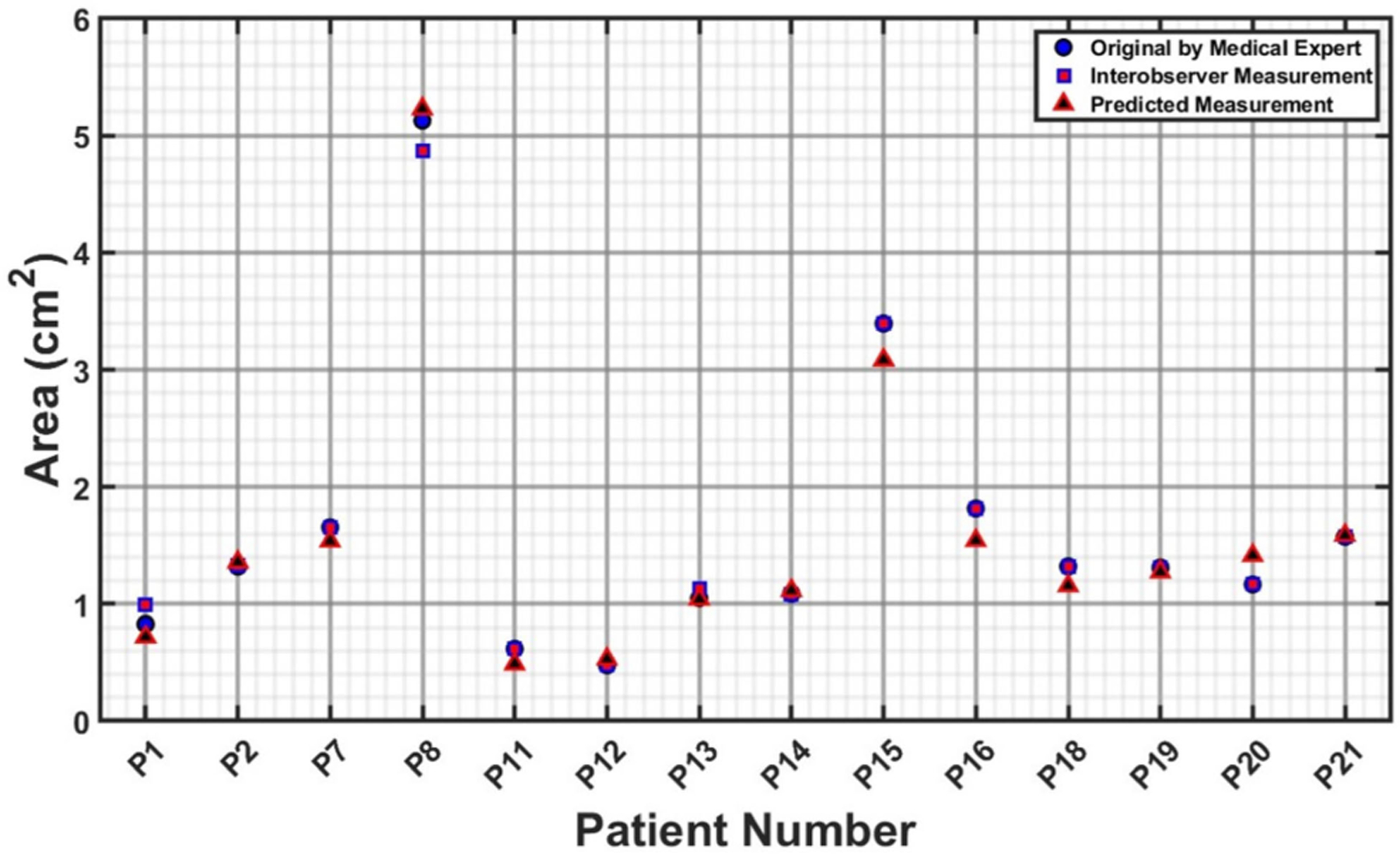
Comparative analysis of lumen area of the right common iliac artery (in cm^2^).

The measurements illustrated in Figures 6–9 are based on the four cross sections of clinical relevance illustrated in Figure 5 and include the original measurements resulting from segmentations performed by a medical expert, by interobserver variability, and by automated model prediction. Figures 6, 8, and 9 show a comparative analysis of lumen area of the neck, left common iliac artery, and the right common iliac artery, respectively. Not all of the 22 patients had a well-defined neck or available CTA images of the left and right common iliac arteries. Figure 7 shows a comparative analysis of the maximum aneurysm diameter (measured using the hydraulic diameter definition) based on the outer wall boundary. Although the aforementioned performance metrics are valuable for assessing the model’s segmentation performance, a comparison of clinically relevant measures with the ground truth would yield the translational significance of having an automated segmentation tool for the management of AAAs in the clinic. To this end, the average percentage difference in the predicted neck lumen area and the original neck lumen area measured by a medical expert was 5.1% for all patients shown in Figure 6. Similarly, the average percentage difference between the predicted and original maximum aneurysm diameter was 8.3% for all patients shown in Figure 7. Furthermore, the average percentage differences in the left and right iliac artery lumen areas (predicted vs. original) were 8.8% and 8.7%, respectively, for the patients shown in Figures 8 and 9. The systematic comparison between multiple patient profiles and varied anatomical features, as depicted in these figures, supports the applicability and robustness of the method.

**FIGURE 10.**
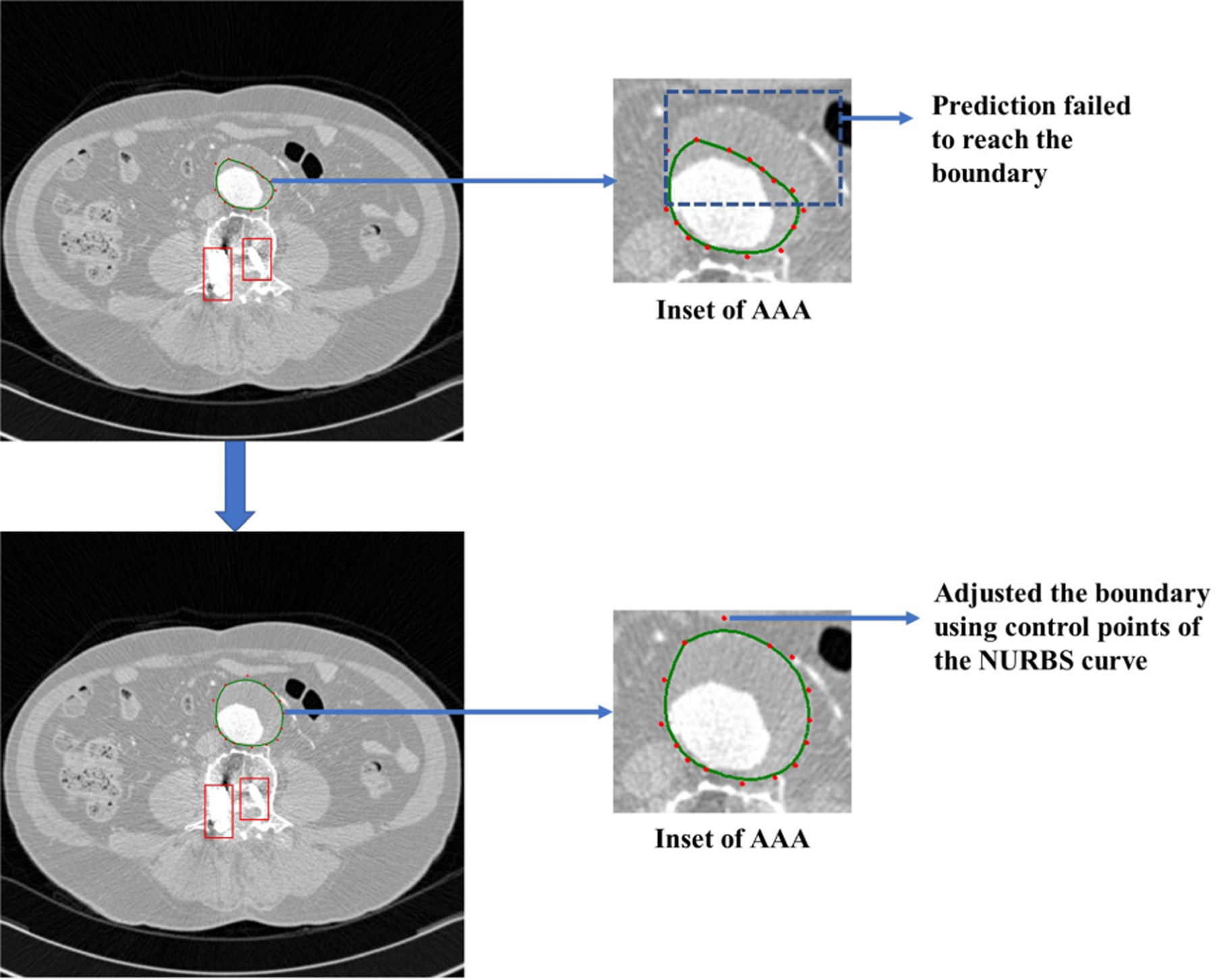
Segmentation of a complex AAA case with metal artifacts (shown in red boxes): (a) Failure of the automated prediction algorithm and (b) Corrected segmentation achieved using NURBS.

In a complex case of an AAA patient under surveillance, CT images at a follow-up revealed a metal artifact (a screw in the spinal column), which resulted in a failure of the automated prediction to accurately outline the outer wall boundary. This follow-up CTA exam consisted of 142 images, with the prediction algorithm failing to segment the outer wall in 6 of these images. Figure 10 shows the automated segmentation failure caused by metal artifacts (shown enclosed by the red boxes) and the corrected segmentation achieved using control points of the NURBS curve. To enhance the accuracy of the segmentation, NURBS were employed to manually adjust the points, thereby ensuring that the identified outer wall boundary yields a clinically relevant accuracy.

## V. DISCUSSION

In routine clinical practice, the size assessment of an AAA often involves a manual measurement of the maximum aortic diameter, which can be susceptible to interobserver variability. Similarly, the areas of the neck and common iliac arteries are measured as part of a pre-operative planning strategy. In this work, a deep learning-based model was presented for the automatic segmentation of contrast-enhanced abdominal CTA images that correctly identify the lumen and outer wall boundaries of an AAA compared to the ground truth. Segmentation is aided by the implementation of a NURBS-based method, which improves the accuracy of the model and facilitates the correction of complex anatomical cases, especially when metal artifacts are present in the images. The quantitative analysis and performance metrics of the model provide evidence of its accuracy and potential use as part of an integrated pipeline for the risk assessment of AAAs in the clinic.

The fully automated model outperforms other deep learning methods, as summarized in Table 2, with exceptional DSC scores of 95.62 and 96.58 for the lumen and outer wall segmentation, respectively. It should be noted that the same test dataset was not used for all comparisons in Table 2. The key feature of the model is the strategic use of a patch-based dilated U-Net architecture, addressing the critical limitations observed in previous CNN models. Using a patch-based approach, the proposed method efficiently leverages training data without overfitting, a common concern in segmentation tasks. Furthermore, the unique configuration, including a non-overlapping patch strategy implemented by aligning the stride with the patch size, maximizes accuracy without undue computational burden. An additional enhancement involves the incorporation of dilation parameters in the CNN, which significantly increases the receptive field while maintaining computational efficiency through consistent filter sizes. In particular, the model exhibits good agreement with ground-truth data, particularly in cases with complex tortuosity. It also has exceptional speed, measured at17 ± 0.02 milliseconds per image to generate the final segmented output.

Lareyre et al. [48] recently introduced an automated pipeline designed to detect the AAA lumen during segmentation. Their methodology incorporates feature-based techniques, employing boundary propagation and active contour methods to precisely segment the aortic lumen. This approach was validated on a dataset comprising 40 CTAs and showcased commendable performance metrics. Notably, the computational time per patient remained under 1 minute, a significant advantage in a clinical setting. Nevertheless, six cases presented false positives, while six others depicted false negative portions, failing to detect the aorta or the branching of a renal artery.

The use of NURBS in the proposed model was first tested on a dataset comprising CTA images and their corresponding segmentation masks. Users were able to navigate through the images and interactively modify the segmentation boundaries to the contours. The NURBS curves provided a robust mechanism for anatomically accurate representations of the segmentation boundaries. The modified segmentation masks revealed a closer alignment with the actual anatomical structures based on ground-truth segmentations. Furthermore, the use of NURBS significantly reduced the segmentation time. This improvement not only increases the efficiency of the segmentation process, but also makes it more practical for real-time clinical applications, where fast and accurate results are needed for time-sensitive patient care.

An inherent limitation of the present work lies in the model’s dependency on contrast-enhanced CTA images. To moderate this constraint, future work includes integrating unenhanced images into the training dataset once an adequate volume of patient data with such images becomes available. Prospective acquisition of unenhanced images is important, given that a subset of AAA patients experience delayed adverse reactions to radiographic contrast media, ranging from 1% to 23%, occasionally increasing to moderate or severe levels [49]. Moreover, future enhancements will augment the dataset with more diverse CTA images. This expansion in training data holds promise for refining the performance of the model in varying clinical scenarios and anatomical presentations. In addition, there is an imperative need for the identification of the inner wall boundary in the CTA images, a challenging task that has not yet been addressed by previous models without user intervention.

## VI. CONCLUSION

The present work addresses the need for automation and accurate AAA segmentation of contrast-enhanced CTA images. The proposed framework utilizes a patch-based dilated U-Net architecture, overcoming the critical limitations inherent in previous CNN models. This resulted in a method that exhibits high accuracy in identifying the AAA lumen and outer wall boundaries. Through rigorous testing, the model consistently delivers precise segmentation while maintaining a processing time of 17 ± 0.02 milliseconds per image. The combined accuracy and speed can be considered advantageous for expediting clinical decision-making related to AAA management if the tool was used in the clinic. Compared to measurements obtained from different observers (i.e., interobserver variability), the model’s predictions exhibit a notably close alignment with the original measurements, showing its reliability and reproducibility. To further enhance its utility, we integrated NURBS, enabling precise modifications and offering simple user interaction when required. This combination of advanced deep learning with NURBS provides a robust, user-adaptive solution that proves effective in anatomically complex cases. This framework can serve as a foundation for broader applications, including segmentation and biomechanical estimation of other soft tissue structures in CTA imaging. Its adaptability and performance highlight its potential to improve clinical workflows and patient outcomes in AAA management.

## Figures and Tables

**Table 1:** . Performance evaluation based on image segmentation of 18 non-complex AAA cases and 4 complex AAA cases (with high tortuosity).

Metric	Lumen (non-complex AAAs)	Outer Wall (non-complex AAAs)	Lumen (complex AAAs)	Outer Wall (complex AAAs)
Accuracy	0.9995	0.9992	0.9995	0.9994
Sensitivity	0.9482	0.9666	0.9587	0.9695
Precision	0.9667	0.9674	0.9660	0.9785
MCC	0.9566	0.9660	0.9617	0.9736
DSC Coeff.	0.9562	0.9658	0.9616	0.9737
Specificity	0.9998	0.9996	0.9998	0.9997
IOU-score	0.9175	0.9354	0.9269	0.9492

**Table 2.** A summary of segmentation models and their DSC scores.

Classes	Models	DSC Score
Lumen	Graph-Cuts [37]	42.6 ± 4.60
	3DUNet [38]	64.3 ± 5.60
	Segnet [39]	66.3 ± 5.20
	3DResUNet [40]	74.2 ± 4.10
	KiU-Net [41]	70.5 ± 5.00
	CACU-Net [24]	91.6 ± 2.90
	ARU-NET [25]	91.6 ± 2.80
	CNN [26]	95.0
	CNN [29]	92.0 ± 1.00
	Ours	95.62

Outer wall	Intensity-based [42]	94.69 ± 3.54
	Active-contour [43]	52.24 ± 9.84
	Distance Regularized [44]	59.92 ± 10.54
	Graph Cut [45]	68.71 ± 19.35
	Graph cut based [46]	64.86 ± 16.83
	CNN [47]	85.0
	Ours	96.58

Aortic Aneurysm	Intensity-based [35]	90.11 ± 5.97
	Active-contour [44]	35.99 ± 9.70
	Distance Regularized [42]	43.64 ± 11.53
	Graph Cut [44]: 55.62 ± 23.01	
	Graph cut based [45]	50.12 ± 17.75
	3DUNet [48]	90.3 ± 3.15
